# Emotional Eating and Dietary Patterns: Reflecting Food Choices in People with and without Abdominal Obesity

**DOI:** 10.3390/nu14071371

**Published:** 2022-03-25

**Authors:** Alejandra Betancourt-Núñez, Nathaly Torres-Castillo, Erika Martínez-López, César O. De Loera-Rodríguez, Elvira Durán-Barajas, Fabiola Márquez-Sandoval, María Fernanda Bernal-Orozco, Marta Garaulet, Barbara Vizmanos

**Affiliations:** 1 Instituto de Nutrigenética y Nutrigenómica, Centro Universitario de Ciencias de la Salud (CUCS), Universidad de Guadalajara (UdeG), Guadalajara 44340, Mexico; alejandra.bnunez@academicos.udg.mx (A.B.-N.); nathaly.torrescas@academicos.udg.mx (N.T.-C.); erikamtz27@yahoo.com.mx (E.M.-L.); yolanda.marquez@academicos.udg.mx (F.M.-S.); fernanda.bernal@academicos.udg.mx (M.F.B.-O.); 2 Departamento de Disciplinas Filosófico, Metodológicas e Instrumentales, CUCS, UdeG, Guadalajara 44340, Mexico; 3 Doctorado en Ciencias de la Nutrición Traslacional, CUCS, UdeG, Guadalajara 44340, Mexico; 4 Departamento de Biología Molecular y Genómica, Centro Universitario de Ciencias de la Salud, Universidad de Guadalajara, Guadalajara 44340, Mexico; 5 Departamento de Fisiología, Centro Universitario de Ciencias de la Salud, Universidad de Guadalajara, Guadalajara 44340, Mexico; cesar.deloera@redudg.udg.mx; 6 Coordinación General de Recursos Humanos, Universidad de Guadalajara, Guadalajara 44100, Mexico; elvira.duran@redudg.udg.mx; 7 Departamento de Clínicas de la Reproducción Humana, Crecimiento y Desarrollo Infantil, CUCS, UdeG, Guadalajara 44280, Mexico; 8 Department of Physiology, Regional Campus of International Excellence, University of Murcia, 30100 Murcia, Spain; 9 Biomedical Research Institute of Murcia, IMIB-Arrixaca-UMU, University Clinical Hospital, 30120 Murcia, Spain; 10 Division of Sleep and Circadian Disorders, Brigham and Women’s Hospital, Boston, MA 02115, USA; 11 Division of Sleep Medicine, Harvard Medical School, Boston, MA 02115, USA

**Keywords:** emotional eating, dietary patterns, abdominal obesity, eating style, eating behavior

## Abstract

Emotional eating (EE) is food consumption in response to feelings rather than hunger. EE is related to unhealthy food intake and abdominal obesity (AO). However, little evidence exists about the association between EE and dietary patterns (DPs) and EE–AO interaction related to DPs. DPs allow describing food combinations that people usually eat. We analyzed the association of EE with DPs in adults (≥18 years) with AO (WC ≥ 80/90 cm in women/men, respectively; *n* = 494; 66.8% women;) or without AO (*n* = 269; 74.2% women) in a cross-sectional study. Principal component analysis allowed identifying four DPs from 40 food groups (validated with a semiquantitative food frequency questionnaire). Among the subjects presenting AO, being “emotional/very-emotional eater” (emotional eating questionnaire) was negatively associated with the “Healthy” DP (fruits, vegetables, olive oil, oilseeds, legumes, fish, seafood) (OR:0.53; 95% CI: 0.33, 0.88, *p* = 0.013) and positively with the “Snacks and fast food” DP (sweet bread, breakfast cereal, corn, potato, desserts, sweets, sugar, fast food) (OR:1.88; 95% CI: 1.17, 3.03, *p* = 0.010). Emotional eaters with AO have significantly lower fiber intake, folic acid, magnesium, potassium, vitamin B1, and vitamin C, while they had a higher intake of sodium, lipids, mono and polyunsaturated fatty acids, and saturated fats. In non-AO participants, EE was not associated with any DP (*p* > 0.05). In conclusion, EE is associated with unhealthy DPs in subjects with AO.

## 1. Introduction

Environmental and individual determinants influence food consumption [1,2]. Personal aspects include emotions, which can influence food intake [1,2,3,4]. Negative emotions produce a series of physiological reactions that naturally promote a lack of appetite or decreased food intake [3,5]. However, some people increase their food intake in response to negative emotions such as emotional stress, anxiety, frustration, sadness, anger, and loneliness [3]. This situation is known as “emotional eating” [3,5,6]. It has been evidenced that, on average, 30% of people increase and 48% decrease their appetite or food intake when facing a negative emotion, such as emotional stress [3].

Several studies have assessed the association of emotional eating with energy and macronutrient intake or particular food choices [3,7,8,9,10,11,12,13,14]. Emotional eating has been associated with fast food intake [10,12], salty snacks [9,10], sweet high-fat foods [3,7], or energy-dense foods [9] such as cakes, biscuits, pastries [7,8], ice cream, chocolate and its products, breakfast cereals [7], candies [11], and artificially sweetened beverages [7,9,11]. Furthermore, emotional eating has been positively associated with waist circumference (WC) [15,16], abdominal obesity (AO) by WC [17,18], body mass index (BMI) [15,16,19,20], obesity by BMI [6,12,17], obesity by percent body fat [18], bodyweight gain [6,21], and weight loss impairments [21]. Moreover, emotional eating has been shown to interact with the physiological and genetic characteristics of the individual for body weight loss effectiveness [22,23]. In addition, obesity and AO are more frequent among those subjects classified as emotional eaters than non-emotional ones [17].

Despite this relatively large number of studies, few studies have analyzed the potential association between emotional eating and specific dietary patterns (DPs) [15,24,25,26]. Only one study evaluated the interaction between emotional eating and obesity/abdominal obesity with specific DPs, with non-significant results [15]. Assessing DPs is interesting because it allows for the description of the complete diet, i.e., the food combinations people usually eat [27]. This diet description is close to reality since people do not typically consume individual foods or nutrients. On the other hand, considering that emotional eating is a risk factor for developing obesity [6,12,15,16,17,18,19,20], the interest arises to analyze whether emotional eating is associated with specific DPs in people with and without obesity.

While BMI is widely used as an index of obesity, it does not differentiate between body lean mass and body fat mass and does not reflect the location of body fat [28]. Instead, WC is an indicator of abdominal fat that has been associated with increased cardiometabolic risk [29,30]. Therefore, considering the advantage of measuring AO with WC vs. BMI, we analyzed if emotional eating relates to the DPs in people with and without AO.

Identifying the different DPs in people with and without AO and looking for potential connections with emotional eating is essential in designing strategies to improve adherence to a healthy DP and prevent and treat AO. We aimed to analyze the association of emotional eating with various DPs in adults with and without AO.

## 2. Materials and Methods

### 2.1. Characteristics of the Participants

The present article is part of the macro project entitled “Association of Diet Quality Index, emotions and chronotype with Body Mass Index in workers of the University of Guadalajara (CADICEM)” within this university’s “Organizational Health Program” framework. The University of Guadalajara has 15 University Centers spread throughout the state of Jalisco (78 thousand km^2^), Mexico: of those, 14 participated in this macro project. The university also has a virtual university system and a high school system: both were also asked to join at the end of 2019. The participants’ selection was non-random; all the workers were verbally invited during a visit to their workplace. This macro project included volunteers, men or women over 18 years of age, among workers of this public university in Mexico. Pregnant women were not involved in the study.

**Figure 1:**
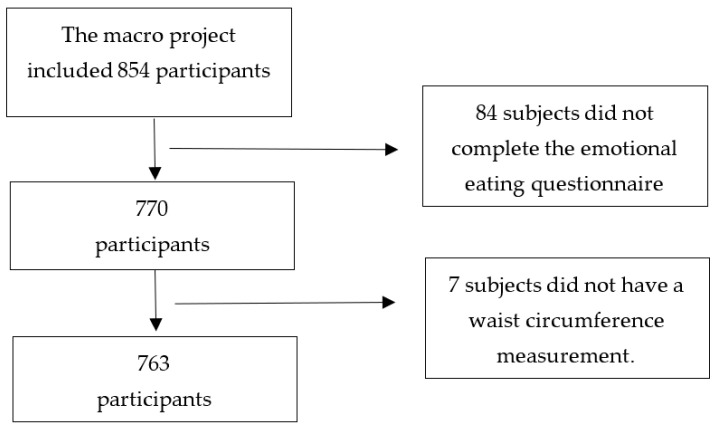
Flow chart.

For the present study, all participants of the macro project with WC determination and who reported sociodemographic, dietary, and emotional eating characteristics were included. The variables analyzed for this study are described below. In this sense, the macro project had 854 participants; however, for the present study, we eliminated 84 subjects because they did not complete the emotional eating questionnaire, and 7 participants did not have their WC measurement. The 91 people excluded were female (64.8%), older than 31 years (71.4%), administrative workers (64.4%), sedentary (71.4%), and having AO (65.1%) (in those who had their WC measurement). The present study included 763 participants (Figure 1).

This project was approved by the Research Ethics, Research and Biosafety Committees of the Centro Universitario de Ciencias de la Salud, Universidad de Guadalajara (CI-02719) and was performed according to the last version of the Declaration of Helsinki [31]. After opening a dialogue to clarify doubts regarding their participation, all participants voluntarily signed an informed consent letter.

### 2.2. Sociodemographic Characteristics

Participants were asked about their age, sex, occupation (academic, managerial, operative, administrative) and perception of physical activity (sedentary, active, or very active) by employing an interview. Additionally, we recorded the University Center to which they were attached.

### 2.3. Anthropometric Measurements

Bodyweight (Tanita^®^ BC-568 electric scale, precision 0.1 kg) and height (portable stadiometer Seca 213^®^, precision 0.1 cm) were measured by a nutritionist to calculate the BMI. Waist circumference was measured (fiberglass tape measure) at the midpoint between the edge of the lower costal (tenth rib) and the iliac crest, after one exhalation, following normal breathing [32]. Individuals were classified using WC into AO (≥80 cm in women and ≥90 cm in men) and non-AO groups (<80 cm in women and <90 cm in men) according to the specific cut-off points for the Central and South American population proposed by Alberti et al., 2009 [33].

### 2.4. Emotional Eating

Participants completed the self-reported emotional eating questionnaire (EEQ), validated in Spain in adults with obesity [34] and in university students with and without obesity [35]. This questionnaire contains ten questions, with four response options that have a specific score: never (0 points), sometimes (1 point), usually (2 points), and always (3 points). The total score (sum of each question score) allows the participants to be classified as non-emotional eaters (0–5 points), low emotional eaters (6 to 10 points), and emotional or very emotional eaters (11 to 30 points). For the current study, the EEQ was analyzed by academics and graduate students. They perceived it was adequately understood and did not find words not recognized in the Mexican lexicon. Therefore, we decided to apply it without any changes. We evaluated its reliability (internal consistency). Cronbach’s alpha for the entire instrument was 0.838 when including all participants, 0.851 when including only participants with AO, and 0.761 when including participants with non-AO. These Cronbach’s alpha values are interpreted as satisfactory [36].

### 2.5. Dietary Intake

A semiquantitative food frequency questionnaire (SFFQ) of 162 items, previously validated in the Mexican population, was used [37]. A trained nutritionist team applied it by interview, using a validated Mexican photo album as visual support [38]. Participants mentioned each food item’s frequency and regular consumption in the year before the survey. From this information, the nutritionist recorded the number of servings consumed of each food (each item has a standard serving), choosing from nine possible response options ranging from never or seldom to more than six servings per day.

From the number of servings consumed and the standard portion of each item, we calculated each participant’s daily nutrition intake of the 162 items. The daily intake of all items was then grouped into 40 food groups (Table 1) according to their nutritional characteristics (carbohydrate, protein, lipid, sugar, fiber, and alcohol content) and preparation (industrialized or home-prepared foods). Some foods were not grouped and formed a food group by themselves.

The responses recorded in the SFFQ were captured in the Nutricloud^®^ software (https://www.nutricloud.mx/, accessed on 17 February 2022, software as a service, Guadalajara, Mexico) to determine energy and nutrient intake data. This software included Mexican food composition data [39] and the USDA [40].

### 2.6. Statistical Analysis

Qualitative variables were expressed as frequency and percentage, while quantitative variables were expressed as mean and standard deviation. We analyzed the association between qualitative variables with the Chi^2^ test. Student’s t-test was used to compare quantitative variables between those subjects with and without AO. We compared quantitative variables among the three groups of emotional eaters (non-emotional eaters, low emotional eaters, and emotional/very emotional eaters) with one-factor ANOVA and ANCOVA adjusted by energy intake. Bonferroni’s post hoc statistical tests were carried out between the groups of non-emotional with emotional/very emotional eaters, when they achieved significant differences. Those variables with non-normal distribution were log-transformed. However, the mean values are shown in the original variables.

We generated DPs using principal component analysis (PCA) from the 40 food groups in the total sample. Before performing this statistical approach, Kaiser-Meyer-Olkin analysis and Bartlett’s sphericity test were performed to define the feasibility of multivariate analysis with these variables. We interpreted the results of the Kaiser-Meyer-Olkin analysis (0.70) and the Bartlet sphericity test (*p* < 0.001) as acceptable; therefore, it was feasible to perform the multivariate analysis [41,42]. The first four components were selected and named “Snacks and fast food,” “Traditional Westernized”, “Healthy,” and “Animal products and cereals” (Supplementary Figure S1 and Supplementary Table S1). The number of DPs was defined with the help of the scree plot test [41,42,43]. We applied a Varimax rotation analysis to improve factor loadings interpretation and obtain uncorrelated components [41,42]. As part of a DP, a factor loading between each food group, equal or greater than 0.3 (positive or negative), was considered significant [43] and maintained in the model. When the factor loadings were between 0.25 to 0.3 (positive or negative), the food groups remained in the model because they were considered relevant in the conformation of the DP. If a food group had a factor loading ≥0.3 in more than one DP, this food group was considered part of more than one DP. Food groups that presented low factor loadings (≤0.25) in the four DP were eliminated from the PCA. For this reason, the PCA performed in the total sample did not include coffee, natural water, and skim milk.

We calculated the potential interaction between emotional eating and obesity (classified by BMI) with the four DPs identified, adjusted by WC, but no significant interaction was observed. Nevertheless, a significant interaction was observed between emotional eating and AO with the “Healthy” DP (*p* = 0.046), adjusted by BMI. Thus, we decided to generate DPs in participants with and without AO following the abovementioned process.

The results of the Kaiser-Meyer-Olkin analysis (0.67 in participants with non-AO, and 0.75 in participants with AO) and the Bartlet sphericity test (*p* < 0.001) were interpreted as acceptable [41,42]. The first four components were selected. The DPs in non-AO participants did not contain salt or skim milk, and the DPs in those with AO did not include natural water or coffee. Because these food groups presented low factor loadings (≤0.25) in the four DPs, we eliminated them from the PCA.

The factor scores for each DP were divided into two categories (50th percentile). The lower half (lower score) was interpreted as non-adherence to the DP, and the upper half (higher score) was interpreted as adherence to the DP. All the participants had a score in each of the DPs; hence, each subject could score high in more than one DP. We assessed the association of DPs (dependent variable) with the emotional eater classification (independent variable) with multivariate logistic regression analyses, adjusted for age, BMI, sex, energy intake, and physical activity. These associations were performed according to whether AO or non-AO was present. A value of *p* ≤ 0.05 was considered significant. We developed all analyses with IBM SPSS Statistics for Windows, version 28 (IBM Corp., Armonk, NY, USA).

## 3. Results

### 3.1. General Characteristics of the Participants

The present study included 494 participants with AO (64.7%) and 269 without AO (35.3%). Their characteristics are shown in Table 2. Compared to those with non-AO, those with AO were emotional or very emotional eaters in a higher frequency, were older, presented higher BMI, and were more sedentary (Table 2). Emotional eaters in the AO group had a higher BMI and were more sedentary than non-emotional eaters. Furthermore, within the AO and non-AO groups, a higher proportion of women were among the emotional or very emotional eaters (Table 2). We found no significant differences in the occupation between AO and non-AO subjects or across the different emotional eater categories.

### 3.2. Description of the Dietary Patterns in Participants with and without Abdominal Obesity

**Figure 2:**
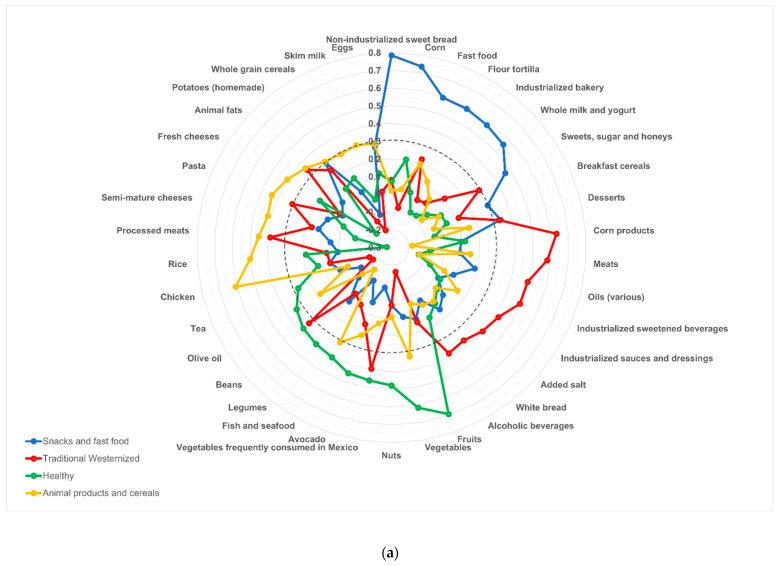

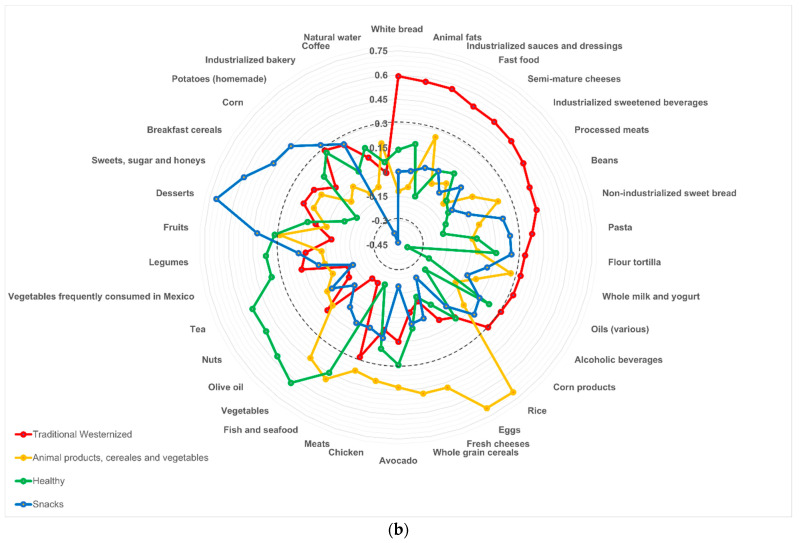
Dietary patterns drawn from participants with and without abdominal obesity. This figure shows the dietary patterns (DPs) identified in participants with abdominal obesity (**a**), and those without abdominal obesity (**b**). The numbers expressed are the factor loads obtained by principal component analysis. DPs are only made up of food groups with a factor load equal to or greater than 0.3 or close to 0.3 (positive or negative). The dotted line indicates the factorial load equal to 0.3 to improve the interpretation of the graph. If a food group had a factor loading ≥0.3 on more than one DP, this food group was considered part of more than one DP. The food groups that make up the DP named “Traditional Westernized” are in red. The food groups that make up the DP named “Animal products and cereals” or “Animal products, cereals, and vegetables” are drawn in yellow. The green color shows the food groups that make up the DP named “Healthy”, and the blue color shows the food groups that make up the DP named “Snacks and fast food” or “Snacks” in the abdominal obesity group and the non-abdominal obesity, respectively.

Figure 2 shows the four DPs identified in those with and without AO. In those who presented AO, the first DP, which explained the most significant percentage of the variance (12.5% of the total variance, Supplementary Table S1), was named “Snacks and fast food” because it was made up of non-industrialized sweet bread (with factor load of 0.785) followed by corn, fast food, flour *tortilla*, industrialized bakery, sweets and sugar, whole milk and yogurt, desserts, potato, and breakfast cereals. The second DP (7.06% of the total variance, Supplementary Table S1) was named “Traditional Westernized” because it was made up of food groups traditionally consumed in Mexico (corn products like *tortilla*, meat, oil, vegetables frequently consumed in Mexico, alcoholic beverages, white bread, animal fat, and beans), together with foods characteristic of western culture (industrialized sweetened beverages, industrialized sauces and dressings, added salt, processed meats, desserts, and pasta). The third DP (5.71% of the total variance, Supplementary Table S1) was named “Healthy” because it consisted of the following food groups: fruits (with factor load of 0.69) followed by vegetables, nuts, avocado, fish and seafood, legumes, beans, olive oil, and tea. Finally, the fourth DP (4.39% of the total variance, Supplementary Table S1) was named “Animal products and cereals” because it consisted of chicken (with factor load of 0.6) followed by rice, pasta, semimature cheeses, fresh cheeses, whole grain cereals, skim milk, and eggs (Figure 2a).

In non-AO subjects, the first DP, which explained the most significant percentage of the variance (11.13%, Supplementary Table S1), was the “Traditional Westernized” DP. This pattern received this name (similar to the one for subjects with AO) because it was made up of food typical of the Mexican food culture (white bread, animal fat, beans, non-industrialized sweet bread, flour *tortillas*, oils, alcoholic beverages, and corn products), in combination with foods from the western culture (industrialized sauces and dressings, fast food, semimature cheeses, industrialized sweetened beverages, processed meats, pasta, whole milk, and yogurt). The second DP (8.53% of the total variance, Supplementary Table S1) included animal products (eggs, fish and seafood, fresh cheeses, chicken, meat), cereals (rice, whole grains), and vegetables (vegetables, avocado). Therefore, this DP was named “Animal products, cereals, and vegetables.” The third DP (5.75% of the total variance, Supplementary Table S1) was also called “Healthy” because it included vegetables, olive oil, tea, nuts, fish and seafood, legumes, and fruits with low whole milk and yogurt intake. Finally, the “Snacks” DP (4.82% of the total variance, Supplementary Table S1) included desserts, sweets and sugar, breakfast cereals, corn, fruits, potatoes, and industrialized bakery products with a low intake of natural water and coffee (Figure 2b).

Supplementary Table S1 shows the factorial loads of the food groups in each DP and the percentage of the total variance of each DP. Supplementary Tables S2 and S3 show energy and nutrient intake according to adherence to DPs in participants with and without AO, respectively.

Participants with/without AO (Table 3) who adhered to any DP consumed significantly more energy than the non-adherent ones. Similarly, those who stuck to the “Traditional Westernized” DP were characterized mainly as men. Among those who presented AO (Table 3), participants who adhered to the “Snacks and fast food” DP and “Animal products and cereals” DP were younger than those who did not adhere to these DPs. Mainly, those who adhered to the “Healthy” DP were older and more “active or very active” than those who did not comply with this DP. Notably, among subjects without AO (Table 3), those who adhered to the “Traditional Westernized” DP were younger than those who did not adhere to this DP. Likewise, those who stuck to the “Animal products, cereals, and vegetables” DP were characterized by being “very active” regarding physical activity, compared to those non-adherents. 

### 3.3. Adherence to Dietary Patterns According to the Emotional Eating Classification in Participants with and without Abdominal Obesity

**Figure 3:**
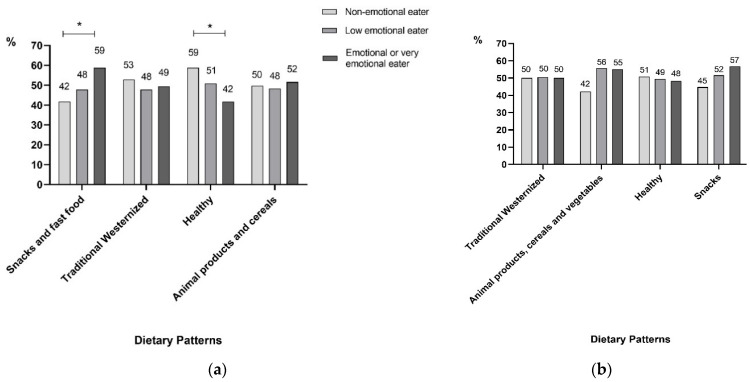
Adherence to dietary patterns according to the emotional eating classification in participants with and without abdominal obesity. This figure shows the adherence to dietary patterns according to the emotional eating classification in participants (**a**) with abdominal obesity or (**b**) without abdominal obesity. The emotional eating classification is presented in three categories: light gray for the non-emotional eaters, medium gray for the low emotional eaters, and dark gray for the emotional or very emotional eaters. The percentages expressed the proportion of subjects in the respective emotional category who adhered to each dietary pattern. It is important to notice that all subjects in a group (with abdominal obesity or without abdominal obesity) adhered or not to the four dietary patterns. * *p* < 0.05.

Importantly, subjects with AO who were emotional or very emotional eaters adhered more to the “Snacks and fast food” DP (*p* = 0.007) and less to the “Healthy” DP (*p* = 0.008) than those who were non-emotional or low emotional eaters (Figure 3a). In subjects with non-AO, significant differences were not found (Figure 3b).

### 3.4. Association between Dietary Patterns and Emotional Eating Classification

After adjustment by age, sex, energy intake, physical activity, and BMI, in subjects with AO, being an emotional or very emotional eater (independent variable) was negatively associated with the “Healthy” DP (OR: 0.54; 95% CI: 0.33, 0.90) and positively associated with the “Snacks and fast food” DP (OR: 1.95; 95% CI: 1.19, 3.18) as opposed to being a non-emotional eater (Table 4). On the other hand, among non-AO subjects, no significant associations were observed between emotional eating and DPs (Table 4).

These results complement and match nutrient intake among the different categories of emotional eaters within each population group. According to the emotional eater classification, in subjects without AO, no significant differences were observed in energy, macronutrients, vitamins, and minerals. Nevertheless, in participants with AO, the emotional eaters consumed significantly less fiber, magnesium, potassium, vitamin B1, vitamin C, and folate, and more lipids, saturated fatty acids, monounsaturated fatty acids, polyunsaturated fatty acids, and sodium than the non-emotional eaters (Table 5). Supplementary Table S4 shows the intake of energy and nutrients in people with and without AO.

## 4. Discussion

The current study was the first to show a significant interaction between AO and emotional eating in relation to different DPs. Having AO and being an emotional or very emotional eater was positively associated with the “Snacks and fast food” DP and negatively with adherence to the “Healthy” DP. Among those non-AO subjects, no significant associations were observed between emotional eating and DPs.

To the best of our knowledge, few studies have described eating as DPs and have evidenced the association between emotional eating and specific DPs [15,24,25,26]. In these few studies, a positive association between emotional eating and DPs constituted by foods rich in sugar or fat was reported. Emotional eating was evaluated by different scales such as the three-factor eating questionnaire [15,25], the emotion-induced eating scale (EIES) [24], and the Dutch eating behavior questionnaire [26], while DPs were generated as in the present study, by principal component analysis. Thus, emotional eating was positively associated with several DPs such as “Energy-dense sweet foods”, “Energy-dense non-sweet foods” [15], “Unhealthy snacks, convenience foods”, “Sweets” [25], and with a DP named “Energy-rich” [26]. These DPs consisted mainly of the following foods: fast food (such as pizza and hamburger), snacks (such as potato chips, French fries, popcorn, ice cream, chocolate, sweets, cakes, pastries, biscuits, and bread), fried foods, and sweetened beverages.

From those studies, only one study showed a significant and negative correlation between the emotional eating score and a healthy DP. This DP was constituted mainly by whole grains, fresh vegetables, fruit, milk, soy products, pork/beef meat, and poultry [26]. In contrast, in two other studies, emotional eating was not significantly related, neither positively nor negatively, to a Mediterranean-type pattern [10,44].

According to the psychosomatic theory, the positive association observed between emotional eating and the “Snacks and fast food” DP and the negative association between emotional eating and the “Healthy” DP could be related to the fact that emotional eaters consume food to reduce the intensity of their negative emotion and to cope with emotions in the absence of another effective strategy [3,4,5]. Hence, it has been evidenced that the consumption of good-tasting foods (usually foods rich in sugar or fat) provides immediate pleasure and reward (positive affective responses) that can decrease the impact of stress [3,5]. In addition, foods induce emotions; therefore, the palatability of these foods, not their nutritional content (rich in carbohydrates or fat), is the main factor in regulating emotions [3,5]. Further, food consumption is considered to distract the person from the experience of the negative emotion [3,5]. Another reason for selecting this type of food in response to an emotional state may reflect food availability and accessibility. Mainly, in the context of the current study, many foods included in the “Snacks and fast food” DP are ready-to-eat and readily available. In contrast, healthy foods, such as fruits and vegetables, are less accessible [45].

We found that the associations between emotional eating and the DPs were significant among subjects with AO. In a study in adults, there was no evidence that a relationship between emotional eating and three DPs (“Sweet foods”, “Non-sweet foods”, and “Fruits and vegetables”) varied with BMI or waist circumference [15].

The obtained results may be related to changes in cortisol levels since cortisol is released in stressful situations (negative emotions). An excess of cortisol has been correlated with an increase in abdominal fat, independent of BMI [46,47,48,49]. Likewise, it has been observed that people with AO, mainly women, in stressful situations, have higher concentrations of cortisol (increased cortisol reactivity) compared to people without AO [47,50]. Furthermore, visceral fat accumulation provides increased intracellular glucocorticoids [47]. High levels of cortisol can increase appetite. Therefore, there could be an increase in caloric intake [47] with a preference for energy-dense foods [46,47,48], specifically foods rich in fat [46,48,51,52], saturated fat [51], and sugar [46,52]. Besides, cortisol levels and the effects of emotional intake (positive or negative) could also be associated with different physical activity patterns. We should explore this to obtain a complete multiparametric picture.

On the other hand, in this study sample, of those subjects who presented AO, 90.3% were overweight or obese as determined by BMI. The psychosomatic theory of obesity proposes that emotions influence food consumption in subjects with and without obesity. In obese subjects, emotions are more closely related to eating [4]. It was suggested that people with obesity have difficulties in differentiating hunger and satiety cues and do not differentiate hunger from other non-pleasurable emotional feelings [4]. In addition, this increased susceptibility may be due to the level of emotional intelligence. One study observed that people with obesity had reduced emotional intelligence, which means that they have difficulties in perceiving, understanding, regulating, and generating emotions for self-control, both in a self-reported way [53,54] and in an implicit way [55]. Thus, emotional intelligence has been negatively associated with emotional eating [56]. One approach that can be applied in interventions in overweight or obese populations to improve emotional eating is mindfulness-based interventions [57].

Among other results identified in the present study that complement the DP results, no significant differences in energy and macronutrient intake were observed between non-emotional eaters and emotional eaters. In this regard, there is no consistent evidence to claim an increase in food intake in emotional circumstances in individuals who score high on self-reported emotional eating [14]. Furthermore, in other studies, no significant associations between emotional eating and macronutrient intake were observed [7,8,13]. However, significant and positive associations between emotional eating and energy intake have been observed in women (with and without depressive symptoms) [7], in men [8], and in both sexes [10]. Nevertheless, in the current study, those subjects with AO who were emotional eaters consumed less fiber, magnesium, potassium, vitamin C, and folate, and more saturated fatty acids than non-emotional eaters. In addition, being an emotional eater and having AO was positively associated with commitment to the “Snacks and fast food” DP and negatively with adherence to the “Healthy” DP. The “Snacks and fast food” DP included foods rich in lipids and saturated fats, such as fast foods, industrialized bakery, and desserts, and was characterized by a lack of fruits, vegetables, legumes, whole grains, and nuts, which are the primary sources of fiber, magnesium, potassium, vitamin B1, vitamin C, and folate. The consumption of these nutrients, which are lacking in this DP, is essential for cardiovascular health. The Dietary Approaches to Stop Hypertension (DASH) promote the intake of fiber, magnesium, potassium, calcium, and vegetable proteins and suggests a lower intake of refined carbohydrates and saturated fat. This DASH diet was associated with systolic and diastolic blood pressure improvements and significant reductions in total cholesterol and LDL concentrations [58].

The present study contributed to scientific knowledge regarding the association of emotional eating and the selection and combination of foods habitually consumed by participants (DP) rather than consumption of individual foods or nutrients. Another strength is that dietary intake was assessed with a validated SFFQ [37]. In addition, this SFFQ was applied by trained nutritionists using visual support [38] to facilitate the transformation process of usual portion sizes consumed by the participants to the portion sizes presented in the SFFQ. This questionnaire refers to identification of food consumption during the year before the survey day; therefore, it allows the estimation of habitual food consumption. Nevertheless, the SFFQ has some limitations. Energy and macronutrient estimation is not as precise as with other methods such as dietary records, because the reported intake is limited to the foods included in the FFQ used. Besides, there may be errors in the estimation of the habitual portion size consumed since it depends on the respondent’s memory. In addition, participants may report dietary intake according to social desirability, which may result in overestimating certain foods and underestimating others [59]. Another limitation is that the information of supplements was not asked, so we may involuntarily underreport the consumption of vitamins and minerals.

One limitation of the present study was its cross-sectional design, so causality cannot be drawn. These results should be tested in study designs with a higher level of scientific evidence. Furthermore, this study was performed before COVID-19, new studies should be performed in the post-pandemic period in which emotions and DPs may dramatically change.

Future lines of research should explore the association between emotional eating, DPs, and psychological factors, such as emotional dysregulation [60], as these factors could become targets for intervention. In addition, other eating styles such as external eating, uncontrolled eating, and restrained eating, and positive emotions could be analyzed in future studies together with DPs due to the scarce evidence in this regard.

## 5. Conclusions

Being an emotional or very emotional eater and presenting AO is related to how we eat and impacts food and nutrient intake. Those subjects who were emotional eaters and had AO adhered more closely to a DP characterized by frequent snacking and fast food (sweet bread, industrialized bakery, flour *tortillas*, sweets, sugar and honey, breakfast cereals, and desserts) and adhered less to a healthy DP (fruits, vegetables, nuts, legumes and beans, olive oil, tea, chicken, and rice, among other food groups). This combination of DPs that characterizes emotional eaters, results in a low-quality diet with a lower intake of fiber, folic acid, magnesium, potassium, and vitamin C, and a higher intake of sodium and saturated fats.

This study was one of the few studies that analyzed the association of emotional eating with specific DPs. In addition, the current study was the first, to the best of our knowledge, to show a significant interaction between emotional eating and AO with different DPs.

Dietary pattern analyses allow describing the complete diet: the combination of foods habitually consumed by participants rather than independent foods or nutrients. Therefore, considering that emotional eating was associated with specific DPs, when implementing individualized interventions to promote adherence to a healthy DP, we suggest evaluating emotional eating mainly in people with AO. For this purpose, it is necessary to strengthen the nutritionist’s training in this area and to collaborate with other health professionals more specialized in these aspects.

## Figures and Tables

**Table 1:** Description of the food groups.

Food Group	Foods Considered in the Group
1. Fish and Seafood	Oysters, squid, crustaceans, white fish, bluefish, salted fish, canned tuna (in oil or water), canned prepared tuna.
2. Meat	Beef, pork, pork rinds, lamb, offal, and liver.
3. Chicken	Chicken with and without skin.
4. Egg	Egg.
5. Semimature cheeses	*Manchego*, Gouda, *Oaxaca* and mozzarella cheeses.
6. Fresh cheeses	*Requesón* (curd), cottage cheese, *panela* cheese, *adobera* cheese.
7. Whole milk and yogurt	Whole milk, evaporated milk, Petit, whole yogurt, and smoothies.
8. Skimmed milk	Skimmed milk.
9. Processed meats	Ham and processed meats.
10. Fast food	Hamburger, industrialized French fries, popcorn, sachet soups.
11. Industrialized sauces and dressings	Mustard, hot sauce, ketchup, mayonnaise.
12. Breakfast cereals	Breakfast cereals.
13. Industrialized bakery	*María* type cookies, chocolate cookies, industrialized bread, vegetable shortening, and margarine.
14. Sweets, sugar, and honey	Honey, jam, candies, fruit in syrup, *ate* (quince paste), *cajeta* (caramel sauce), condensed milk, sugar, and *piloncillo* (brown sugar).
15. Desserts	Custard, chocolate, cocoa, ice cream.
16. Industrialized sweetened beverages	Light soda, regular soda, canned juices, fermented-milk beverages (e.g., *Yakult*).
17. Alcoholic beverages	Distilled spirits, liquor, beer, white wine, rosé wine, young red wine, and aged red wine.
18. Oils (various)	Corn oil, soybean oil, sunflower oil, mixed oils, canola oil, safflower oil.
19. Olive oil	Extra-virgin olive oil, olive oil, olives.
20. Animal fats	Bacon, lard, cream, cream cheese, and butter.
21. Corn products	Corn dough, corn *tortilla*, and toast.
22. Pasta	Pasta: noodle, spaghetti, macaroni.
23. Rice	Rice.
24. White bread	*Bolillo*, white bread, and buns.
25. Whole grain cereals	Whole grain cereals (oatmeal, granola), whole grain crackers, whole wheat bread.
26. Non-industrialized sweet bread	Sweetbread, cake, homemade bread, *mantecada* (shortbread), doughnut, *churro*.
27. Flour *tortilla*	Flour *tortilla*.
28. Vegetables frequently consumed in Mexico	Onion, garlic, tomato, chili, bell pepper, *poblano* chili, lemon
29. Vegetables	Green bean, cabbage, chard, asparagus, herbs, other vegetables, eggplant, *chayote* (squash) and *jicama* (yam bean), carrot and pumpkin flower, lettuce, peas, mushrooms, and *nopales* (prickly pear leaf).
30. Fruits	Orange, kiwi, guava, lime, mango, pineapple, strawberry, plum, prune, *tuna* (prickly pear fruit), grape, peach, watermelon, melon, papaya, apple, raisins, tamarind, banana, dates, prune, natural fruit juices.
31. Avocado	Avocado.
32. *Elote*	Fresh corn.
33. Potatoes	Potatoes prepared homemade.
34. Nuts	Almonds, walnuts, and peanuts.
35. Beans	*Frijoles* and *alubias* (beans and white beans).
36. Leguminous plants	Lentils and chickpeas.
37. Tea	Tea.
38. Coffee	Coffee.
39. Natural water	Natural water.
40. Salt	Salt added.

Some foods are written in italics because we used their Mexican Spanish name.

**Table 2:** Characteristics of the participants with and without abdominal obesity and according to the emotional eating classification.

	Total	Abdominal Obesity	Non-Abdominal Obesity	Abdominal Obesity			Non-Abdominal Obesity		
				Non-EE	Low EE	EE or Very EE	Non-EE	Low-EE	EE or Very EE
*N* (%)	763	494 (64.7)	269 (35.3)	153 (31.0)	159 (32.2)	182 (36.8) ***	114 (42.4)	95 (35.3)	60 (22.3)
Age (years)	38 ± 11	40 ± 11 ***	34 ± 9	42 ± 11	41 ± 11	38 ± 11 ^●^**	36 ± 10	33 ± 9	32 ± 6 ^●^**
BMI (kg/m^2^)	27.3 ± 5.2	29.8 ± 4.6 ***	22.8 ± 2.4	28.7 ± 3.6	29.7 ± 4.4	30.8 ± 5.2 ^●^***	22.4 ± 2.5	23.1 ± 2.4	23.1 ± 2.2
WC (cm)	88.2 ± 13.4	95.1 ± 11 ***	75.5 ± 6.1	94.4 ± 9.7	95.5 ± 11.5	95.4 ± 11.5	76 ± 6.5	75.6 ± 6.2	74.4 ± 4.7
Sex									
Female	530 (69.5)	330 (66.8) *	200 (74.3)	85 (55.6)	102 (64.2)	143 (78.6) ***	78 (68.4)	68 (71.6)	54 (90.0) **
Occupation									
Academic	74 (9.8)	45 (9.3)	29 (10.9)	14 (9.3)	14 (8.9)	17 (9.6)	10 (8.9)	14 (14.9)	5 (8.3) ^+^
Managerial	54 (7.2)	35 (7.2)	19 (7.1)	15 (9.9)	10 (6.4)	10 (5.6)	7 (6.3)	8 (8.5)	4 (6.7)
Administrative	546 (72.6)	348 (71.6)	198 (74.4)	97 (64.2)	118 (75.2)	133 (74.7)	85 (75.9)	65 (69.1)	48 (80.0)
Operative	37 (4.9)	31 (6.4)	6 (2.3)	12 (7.9)	8 (5.1)	11 (6.2)	3 (2.7)	3 (3.2)	0 (0)
Academic and other	41 (5.5)	27 (5.6)	14 (5.3)	13 (8.6)	7 (4.5)	7 (3.9)	7 (6.3)	4 (4.3)	3 (5.0)
Physical activity									
Sedentary	519 (68)	353 (71.5) ***	166 (61.7)	104 (68.0)	108 (67.9)	141 (77.5) *	67 (58.8)	59 (62.1)	40 (66.7)
Active	151 (19.8)	96 (19.4)	55 (20.4)	31 (20.3)	33 (20.8)	32 (17.6)	26 (22.8)	19 (20.0)	10 (16.7)
Very active	93 (12.2)	45 (9.1)	48 (17.8)	18 (11.8)	18 (11.3)	9 (4.9)	21 (18.4)	17 (17.9)	10 (16.7)

EE: Emotional Eater; BMI: Body Mass Index; WC: Waist circumference. Other: administrative, managerial, operational. Data are presented as mean ± standard deviation and *n* (%). Differences between those with abdominal obesity and non-abdominal obesity were calculated by Student’s t-test. Differences between the three emotional eater categories were calculated by one-factor ANOVA. *p* < 0.05 was considered significant. * *p* < 0.05; ** *p* < 0.01; *** *p* < 0.001. Differences between the non-emotional eaters vs. emotional or very emotional eaters were calculated with Bonferroni’s post hoc statistical test, ● *p* < 0.05. Relationship between qualitative variables was evaluated with the Chi^2^ statistical test. ^+^ Not applicable Chi^2^ test, 40% have an expected frequency less than 5.

**Table 3:** Participant characteristics between subjects who adhere or do not adhere to each dietary pattern in participants with or without obesity.

	**Abdominal Obesity (*n* = 494)**							
	**Snacks and Fast Food**		**Traditional Westernized**		**Healthy**		**Animal Products and Cereals**	
	**No Adherence**	**Adherence**	**No Adherence**	**Adherence**	**No Adherence**	**Adherence**	**No Adherence**	**Adherence**
Age (years)	42 ± 11	38 ± 10 ***	40 ± 11	40 ± 11	37 ± 10	43 ± 12 ***	42 ± 11	39 ± 11 **
BMI (kg/m^2^)	29.7 ± 4.3	29.9 ± 4.9	29.3 ± 4.3	30.3 ± 4.8 *	29.9 ± 4.8	29.8 ± 4.3	29.5 ± 4.4	30.1 ± 4.7
WC (cm)	94.9 ± 10.6	95.4 ± 11.3	92.9 ± 10.2	97.4 ± 11.2 ***	94.7 ± 11.3	95.6 ± 10.7	95.0 ± 11.3	95.3 ± 10.7
Energy intake	2058 ± 758	2616 ± 1097 ***	1920 ± 801	2754 ± 971 ***	2104 ± 770	2571 ± 1110 ***	2185 ± 954	2490 ± 989 ***
Sex								
Female	163 (49.4)	167 (50.6)	192 (58.2)	138 (41.8) ***	165 (50.0)	165 (50.0)	163 (49.4)	167 (50.6)
Male	84 (51.2)	80 (48.8)	55 (33.5)	109 (66.5)	82 (50.0)	82 (50.0)	84 (51.2)	80 (48.8)
Occupation								
Academic	26 (57.8)	19 (42.2) *	22 (48.9)	23 (51.1)	22 (48.9)	23 (51.1)	23 (51.1)	22 (48.9)
Managerial	24 (68.6)	11 (31.4)	18 (51.4)	17 (48.6)	15 (42.9)	20 (57.1)	18 (51.4)	17 (48.6)
Administrative	163 (46.8)	185 (53.2)	183 (52.6)	165 (47.4)	177 (50.9)	171 (49.1)	177 (50.9)	171 (49.1)
Operative	13 (41.9)	18 (58.1)	8 (25.8)	23 (74.2)	15 (48.4)	16 (51.6)	17 (54.8)	14 (45.2)
Academic and other	17 (63.0)	10 (37.0)	12 (44.4)	15 (55.6)	16 (59.3)	11 (40.7)	10 (37.0)	17 (63.0)
Physical activity								
Sedentary	174 (49.3)	179 (50.7)	160 (45.3)	193 (54.7) ***	192 (54.4)	161 (45.6) **	168 (47.6)	185 (52.4) *
Active	51 (53.1)	45 (46.9)	65 (67.7)	31 (32.3)	38 (39.6)	58 (60.4)	59 (61.5)	37 (38.5)
Very active	22 (48.9)	23 (51.1)	22 (48.9)	23 (51.1)	17 (37.8)	28 (62.2)	20 (44.4)	25 (55.6)
	**Non-Abdominal Obesity (*n* = 269)**							
	**Traditional Westernized**		**Animal Products, Cereals, and Vegetables**		**Healthy**		**Snacks**	
	**No Adherence**	**Adherence**	**No Adherence**	**Adherence**	**No Adherence**	**Adherence**	**No Adherence**	**Adherence**
Age (years)	36 ± 9	32 ± 8 ***	34 ± 8	34 ± 10	34 ± 9	34 ± 9	34 ± 9	34 ± 9
BMI (kg/m^2^)	23.1 ± 2.4	22.5 ± 2.4	22.7 ± 2.4	22.9 ± 2.4	23.0 ± 2.5	22.7 ± 2.3	22.9 ± 2.4	22.7 ± 2.4
WC (cm)	75.1 ± 5.9	75.9 ± 6.2	75.6 ± 6.2	75.4 ± 5.9	76.0 ± 6.0	75.0 ± 6.1	75.9 ± 6.1	75.1 ± 6.0
Energy intake	1951 ± 647	2744 ± 848 ***	2050 ± 786	2650 ± 811 ***	2192 ± 801	2507 ± 875 ***	2126 ± 819	2574 ± 827 ***
Sex								
Female	112 (56.0)	88 (44.0) ***	105 (52.5)	95 (47.5)	99 (49.5)	101 (50.5)	97 (48.5)	103 (51.5)
Male	22 (31.9)	47 (68.1)	30 (43.5)	39 (56.5)	36 (52.2)	33 (47.8)	38 (55.1)	31 (44.9)
Occupation								
Academic	16 (55.2)	13 (44.8) *	15 (51.7)	14 (48.3)	14 (48.3)	15 (51.7)	17 (58.6)	12 (41.4) **
Managerial	9 (47.4)	10 (52.6)	9 (47.4)	10 (52.6)	9 (47.4)	10 (52.6)	3 (15.8)	16 (84.2)
Administrative	95 (48.0)	103 (52.0)	100 (50.5)	98 (49.5)	101 (51.0)	97 (49.0)	99 (50.0)	99 (50.0)
Operative	1 (16.7)	5 (83.3)	3 (50.0)	3 (50.0)	2 (33.3)	4 (66.7)	3 (50.0)	3 (50.0)
Academic and other	12 (85.7)	2 (14.3)	5 (35.7)	9 (64.3)	8 (57.1)	6 (42.9)	11 (78.6)	3 (21.4)
Physical activity								
Sedentary	80 (48.2)	86 (51.8)	92 (55.4)	74 (44.6) *	88 (53.0)	78 (47.0)	80 (48.2)	86 (51.8)
Active	32 (58.2)	23 (41.8)	26 (47.3)	29 (52.7)	28 (50.9)	27 (49.1)	27 (49.1)	28 (50.9)
Very active	22 (45.8)	26 (54.2)	17 (35.4)	31 (64.6)	19 (39.6)	29 (60.4)	28 (58.3)	20 (41.7)

BMI: Body Mass Index; WC: Waist circumference. Other: administrative, managerial, operational. Data are presented as mean ± standard deviation and *n* (%). Differences in participant characteristics (those who adhere vs. those who do not adhere to each dietary pattern) were calculated by Student’s t-test. We calculated the log of energy for the statistical analysis; however, in the table, we show the original value of this variable. *p* < 0.05 was considered significant. * *p* < 0.05; ** *p* < 0.01; *** *p* < 0.001.

**Table 4:** Association between emotional eating and dietary patterns in participants with and without abdominal obesity.

Dietary Patterns	Abdominal Obesity (*n* = 494)				Non-Abdominal Obesity (*n* = 269)		
	Non-EE (*n* = 153)	Low EE (*n* = 159)	Emotional or Very EE (*n* = 182)	Dietary Patterns	Non-EE (*n* = 114)	Low EE (*n* = 95)	Emotional or Very EE (*n* = 60)
Snacks and fast food DP				Traditional Westernized DP			
Crude	1	1.27 (0.81, 1.99)	1.98 (1.28, 3.07) *	Crude	1	1.02 (0.59, 1.76)	1.00 (0.53, 1.87)
Model I	1	1.25 (0.79, 1.97)	1.83 (1.17, 2.88) *	Model I	1	0.87 (0.49, 1.58)	1.02 (0.52, 1.98)
Model II	1	1.37 (0.85, 2.22)	1.88 (1.17, 3.03) *	Model II	1	0.71 (0.36, 1.38)	0.76 (0.35, 1.65)
Model III	1	1.40 (0.86, 2.26)	1.95 (1.19, 3.18) *	Model III	1	0.77 (0.39, 1.51)	0.86 (0.39, 1.9)
Traditional Westernized DP				Animal products, cereals and vegetables DP			
Crude	1	0.81 (0.52, 1.27)	0.87 (0.56, 1.34)	Crude	1	1.73 (1.00, 3.01) *	1.68 (0.89, 3.15)
Model I	1	0.88 (0.55, 1.39)	1.08 (0.69, 1.71)	Model I	1	1.79 (1.02, 3.15) *	1.89 (0.98, 3.65)
Model II	1	0.95 (0.56, 1.61)	1.00 (0.59, 1.68)	Model II	1	1.79 (0.98, 3.28)	1.77 (0.88, 3.58)
Model III		0.92 (0.54, 1.56)	0.92 (0.54, 1.57)	Model III	1	1.74 (0.94, 3.21)	1.70 (0.83, 3.46)
Healthy DP				Healthy DP			
Crude	1	0.73 (0.46, 1.14)	0.50 (0.32, 0.78) *	Crude	1	0.94 (0.55, 1.63)	0.90 (0.48, 1.69)
Model I	1	0.74 (0.47, 1.17)	0.56 (0.35, 0.89) *	Model I	1	0.97 (0.56, 1.68)	0.91 (0.47, 1.73)
Model II	1	0.75 (0.46, 1.23)	0.53 (0.33, 0.88) *	Model II	1	0.92 (0.52, 1.63)	0.83 (0.42, 1.60)
Model III	1	0.76 (0.46, 1.24)	0.54 (0.33, 0.90) *	Model III	1	0.95 (0.53, 1.69)	0.86 (0.44, 1.69)
Animal products and cereals DP				Snacks DP			
Crude	1	0.95 (0.61, 1.48)	1.08 (0.70, 1.66)	Crude	1	1.32 (0.76, 2.27)	1.61 (0.86, 3.03)
Model I	1	0.92 (0.59, 1.44)	0.96 (0.62, 1.51)	Model I	1	1.34 (0.77, 2.33)	1.59 (0.83, 3.05)
Model II	1	0.95 (0.60, 1.51)	0.96 (0.61, 1.52)	Model II	1	1.28 (0.71, 2.31)	1.47 (0.74, 2.92)
Model III	1	0.92 (0.58, 1.46)	0.89 (0.56, 1.420)	Model III	1	1.33 (0.73, 2.41)	1.54 (0.77, 3.10)

DP: Dietary pattern; EE: Emotional Eater. Data were presented in Odds ratio (95% confidence interval). Adjusted Model I: adjusted for age and sex. Adjusted Model II: adjusted for age, sex, energy intake, and physical activity. Adjusted Model III: adjusted for age, sex, energy intake, physical activity, and body mass index. * The Odds ratio is significantly different from the reference (non-emotional eaters) *p* < 0.05.

**Table 5:** Energy and nutrient intake according to emotional eater classification in participants with/without abdominal obesity.

	Abdominal Obesity (*n* = 494)			Non-Abdominal Obesity (*n* = 269)		
	Non-EE	Low EE	EE or Very EE	Non-EE	Low EE	EE or Very EE
Energy (Kcal)	2400.5 ± 1096.7	2287.4 ± 1018.0	2328.0 ± 841.9	2290.6 ± 819.5	2397.8 ± 901.9	2382.7 ± 837.6
CH (g)	287.3 ± 132.4	268.5 ± 128.7	273.6 ± 108.2	268.0 ± 101.3	273.3 ± 105.8	280.0 ± 122.6
Fiber (g)	22.5 ± 9.9	20.3 ± 10.1	18.9 ± 8.8 ^●^***	20.8 ± 8.0	20.9 ± 8.4	21.2 ± 9.6
Proteins (g)	89.6 ± 42.3	84.6 ± 31.0	85.2 ± 33.0	85.2 ± 32.0	93.8 ± 39.5	92.2 ± 36.6
Lipids (g)	96.4 ± 50.8	95.5 ± 43.9	99.6 ± 39.5 ^●^***	95.3 ± 34.6	103.5 ± 48.8	99.8 ± 34.4
SFA (g)	26.3 ± 16.0	26.3 ± 11.6	28.4 ± 12.8 ^●^***	26.4 ± 11.7	29.7 ± 15.9	29.1 ± 10.8
MFA (g)	32.9 ± 16.9	33.4 ± 16.9	33.5 ± 13.7 ^●^*	33.5 ± 13.5	36.4 ± 20.5	35.7 ± 13.7
PUFA (g)	20.8 ± 13.4	20.8 ± 13.3	22.5 ± 12.7 ^●^*	20.4 ± 9.9	22.1 ± 13.5	19.7 ± 9.8
Cholesterol (mg)	364.2 ± 296.8	354.6 ± 220.0	376.4 ± 239.3	363.3 ± 234.7	441.1 ± 330.5	432.7 ± 255.1
Ethanol (g)	11.1 ± 28.5	8.3 ± 21.3	5.9 ± 11.4	9.0 ± 25.8	6.3 ± 9.6	6.5 ± 7.6
Calcium (mg)	881.8 ± 536.1	790.7 ± 307.4	818.5 ± 348.0	803.2 ± 348.7	902.0 ± 462.7	876.2 ± 340.7
Phosphorus (mg)	1433.6 ± 644.2	1345.4 ± 496.2	1346.7 ± 505.4	1368.7 ± 505.6	1517.3 ± 650.4	1452.3 ± 572.7
Iron (mg)	20.8 ± 9.4	19.3 ± 8.4	19.4 ± 8.8	20.2 ± 8.2	21.6 ± 10.1	20.9 ± 9.2
Magnesium (mg)	466.8 ± 189.3	423.5 ± 170.1	411.4 ± 155.6 ^●^**	434.6 ± 150.7	450.0 ± 170.2	430.3 ± 172.1
Sodium (mg)	1841.3 ± 1227.3	1815.0 ± 982.9	1994.3 ± 1006.3 ^●^*	1840.1 ± 956.4	1988.7 ± 1093.6	2009.3 ± 1047.2
Potassium (mg)	4084.6 ± 1728.0	3750.0 ± 1597.8	3591.6 ± 1426.3 ^●^**	3840.6 ± 1285.5	3956.9 ± 1477.9	4000.2 ± 1545.3
Zinc (mg)	10.5 ± 4.9	10.0 ± 4.3	9.9 ± 4.1	10.0 ± 3.6	10.6 ± 4.6	10.4 ± 4.1
Selenium (mcg)	38.6 ± 16.2	36.3 ± 22.6	37.9 ± 25.7	38.6 ± 19.3	39.5 ± 21.0	43.9 ± 25.1
Vitamin A (mcg)	961.7 ± 647.2	849.6 ± 499.4	872.9 ± 447.0	871.6 ± 354.3	989.7 ± 544.6	1015.2 ± 607.9
Vitamin B1 (mg)	1.9 ± 0.8	1.8 ± 0.8	1.7 ± 0.6 ^●^*	1.8 ± 0.6	1.8 ± 0.7	1.9 ± 0.7
Vitamin B2 (mg)	2.9 ± 2.1	2.8 ± 1.5	2.6 ± 1.4	2.9 ± 2.1	2.9 ± 1.5	3.4 ± 2.3
Vitamin B3 (mg)	22.5 ± 9.7	21.3 ± 9.8	20.7 ± 8.3	22.1 ± 8.4	22.6 ± 8.9	23.2 ± 9.8
Vitamin B6 (mg)	2.2 ± 1.0	2.0 ± 1.0	1.9 ± 0.8	2.1 ± 0.8	2.2 ± 0.9	2.2 ± 1.0
Folate (mcg)	266.8 ± 141.4	243.4 ± 141.6	227.6 ± 113.6 ^●^*	252.4 ± 99.6	273.6 ± 131.8	264.6 ± 140.6
Vitamin B12 (mcg)	7.5 ± 5.4	7.0 ± 4.4	6.7 ± 3.8	6.6 ± 3.8	8.5 ± 8.0	8.1 ± 5.5
Vitamin C (mg)	346.7 ± 225.2	296.3 ± 205.2	268.8 ± 174.2 ^●^***	303.9 ± 160.9	279.5 ± 137.0	309.1 ± 164.2
Vitamin E (mg)	1.0 ± 1.1	1.0 ± 1.4	0.9 ± 1.1	0.8 ± 0.9	0.9 ± 1.1	1.0 ± 1.0

EE: Emotional Eater; CH: Carbohydrates; MFA: Monounsaturated Fatty Acids; PUFA: Polyunsaturated Fatty Acids; SFA: Saturated Fatty Acids. Data are presented as mean ± standard deviation. The log of all these variables was calculated for the statistical analysis; however, in this table we show the original value of the variables. Differences among the three emotional eater categories were calculated by ANCOVA with these transformed variables, adjusted by energy intake. * *p* < 0.05; ** *p* < 0.01; *** *p* < 0.001. Differences between the “non-emotional eaters” and the “emotional or very emotional eaters” groups were calculated with Bonferroni’s post hoc statistical test with these transformed variables. ^●^ *p* < 0.05.

## Data Availability

The data are not publicly available because when we created the research protocol, we did not think to include this point in the informed consent. We also want to propose improvements to the workers’ health care program and continue to explore analyses that will benefit this community and other potential beneficiaries. The data presented in this study are available on request, completely anonymously, from the corresponding authors.

## Supplementary Materials

The following supporting information can be downloaded at: https://www.mdpi.com/article/10.3390/nu14071371/s1, Figure S1: Dietary patterns in the total sample; Table S1: Dietary patterns generated by principal component analysis in the total sample, in participants with non-abdominal obesity and in participants with abdominal obesity; Table S2: Energy and nutrient intake according to non-adherence and adherence to each dietary patterns in participants with abdominal obesity; Table S3: Energy and nutrient intake according to non-adherence and adherence to each dietary patterns in participants without abdominal obesity; Table S4: Energy and nutrient intake in participants with and without abdominal obesity.
