# Supplementary material for: Emotional Eating and Dietary Patterns: Reflecting Food Choices in People with and without Abdominal Obesity
Source: Nutrients. 2022 Mar 25;14(7):1371. doi: 10.3390/nu14071371 (PMC9002960; doi:10.3390/nu14071371)
Supplement: Supplementary file 1 [file nutrients-14-01371-s001.zip › Supplementary Table S1.pdf]

Supplementary Ttable S1. Dietary patterns generated by Principal Component Analysis in the total sample, in participants with non-abdominal obesity and in participants with abdominal obesity

	TOTAL (n= 770)				Non-Abdominal Obesity (n= 269)				Abdominal Obesity (n= 494)			
**Food groups**	**Snacks and fast food**	**Traditional Westernized**	**Healthy**	**Animal products and cereal**	**Traditional Westernized**	**Animal products, cereals and vegetables**	**Healthy**	**Snacks**	**Snacks and fast food**	**Traditional Westernized**	**Healthy**	**Animal products and cereals**
Non-industrialized sweet bread	0.752	0.110	0.048	-0.045	0.431	0.064	-0.166	0.215	0.785	0.081	0.071	0.021
Fast food	0.504	0.250	0.000	0.210	0.523	-0.019	0.069	0.070	0.594	0.226	0.029	0.199
Flour tortilla	0.527	-0.046	-0.038	0.238	0.335	0.040	0.156	0.251	0.591	0.005	-0.075	0.124
Breakfast cereals	0.390	0.029	-0.020	0.072	0.173	0.118	-0.143	0.472	0.293	0.114	0.040	-0.039
Corn	0.674	-0.050	0.225	-0.038	0.076	-0.054	0.175	0.452	0.735	-0.073	0.204	0.032
Industrialized bakery	0.563	0.010	-0.011	-0.022	0.252	-0.087	0.067	0.258	0.576	0.017	-0.072	0.047
Sweets, sugar and honeys	0.458	0.237	0.079	0.007	0.189	0.120	-0.086	0.592	0.468	0.291	0.032	0.020
Whole milk and yogurt	0.482	0.118	-0.105	0.033	0.328	0.266	-0.393	0.116	0.558	0.108	-0.028	-0.069
Desserts	0.388	0.167	0.018	0.179	0.078	0.008	0.128	0.710	0.336	0.327	-0.049	0.153
Potatoes (homemade)	0.331	0.217	0.169	0.237	0.291	0.006	0.273	0.333	0.307	0.254	0.120	0.314
Meats	0.076	0.484	-0.100	0.270	0.283	0.371	-0.191	0.091	0.088	0.583	-0.080	0.148
Oils (various)	0.150	0.466	-0.178	-0.076	0.324	0.073	-0.244	0.015	0.185	0.493	-0.141	-0.138
White bread	0.191	0.444	0.088	0.007	0.593	-0.118	0.139	0.004	0.143	0.365	0.101	0.086

Added salt	0.012	0.361	0.019	0.073	NA	NA	NA	NA	0.095	0.399	0.049	0.038
Industrialized sauces and dressings	0.079	0.428	-0.057	0.255	0.570	0.255	-0.132	0.054	0.015	0.421	0.029	0.146
Alcoholic beverages	0.061	0.298	0.053	0.206	0.306	-0.028	0.219	0.149	0.040	0.380	0.150	0.068
Animal fats	0.142	0.309	-0.097	0.287	0.573	-0.087	0.183	0.013	0.076	0.346	-0.185	0.360
Corn products	0.112	0.626	0.039	-0.158	0.303	0.099	-0.226	0.186	0.094	0.635	0.117	-0.183
Industrialized sweetened beverages	0.109	0.512	-0.086	0.009	0.498	-0.072	-0.046	0.076	0.083	0.492	-0.063	0.026
Beans	-0.002	0.431	0.194	0.012	0.435	0.222	-0.131	0.023	-0.048	0.333	0.376	-0.076
Processed meats	0.042	0.342	-0.287	0.512	0.472	0.096	-0.083	-0.055	0.045	0.386	-0.274	0.452
Vegetables frequently consumed in Mexico	-0.085	0.470	0.447	-0.012	0.167	0.020	0.358	0.058	-0.071	0.393	0.460	0.135
Avocado	-0.012	0.151	0.428	0.245	0.149	0.431	0.292	-0.194	0.027	0.158	0.450	0.224
Tea	-0.012	-0.197	0.321	0.013	-0.113	-0.007	0.534	-0.143	0.016	-0.164	0.275	-0.031
Vegetables	0.003	-0.083	0.698	0.223	-0.187	0.435	0.631	0.036	0.098	-0.160	0.616	0.323
Olive oil	-0.064	-0.101	0.390	0.193	0.146	0.103	0.567	-0.081	-0.095	-0.176	0.339	0.180
Nuts	0.011	-0.015	0.500	0.069	-0.085	0.075	0.526	0.041	0.028	0.027	0.478	0.093
Legumes	0.112	0.041	0.347	-0.045	0.125	0.026	0.371	0.169	0.087	0.030	0.392	-0.143
Fruits	0.149	0.214	0.674	-0.055	-0.035	0.285	0.317	0.425	0.129	0.147	0.693	0.036
Fish and seafood	-0.070	-0.085	0.452	0.396	-0.183	0.494	0.449	0.098	-0.088	0.066	0.405	0.310
Chicken	0.052	-0.026	0.176	0.559	0.083	0.402	0.200	0.133	0.058	0.056	0.127	0.607
Rice	0.030	0.009	0.226	0.574	0.117	0.704	0.126	0.031	0.004	0.067	0.184	0.500
Pasta	0.178	0.245	-0.064	0.404	0.379	0.009	0.036	0.242	0.093	0.311	-0.005	0.437
Semi-mature cheeses	0.159	0.211	-0.078	0.364	0.514	0.032	0.110	-0.039	0.125	0.164	-0.089	0.420
Eggs	0.125	0.018	0.160	0.383	0.077	0.698	-0.030	-0.220	0.277	0.020	0.124	0.293

Whole grain cereals	0.031	-0.098	0.164	0.374	-0.026	0.482	0.073	0.045	0.055	-0.132	0.145	0.300
Fresh cheeses	-0.003	0.105	0.218	0.259	-0.081	0.483	-0.110	0.030	0.028	0.048	0.183	0.402
Natural water	NA	NA	NA	NA	0.001	0.187	0.069	-0.435	NA	NA	NA	NA
Coffee	NA	NA	NA	NA	0.121	-0.071	0.184	-0.373	NA	NA	NA	NA
Skim milk	NA	NA	NA	NA	NA	NA	NA	NA	-0.106	-0.197	-0.015	0.310
Variance (%)	12.06	7.56	5.26	4.29	11.13	8.53	5.75	4.82	12.5	7.06	5.71	4.39

NA: Not applicable.

Dietary patterns were generated with Principal Component Analysis.

Food groups with no factor loading were not included in the principal component analysis because they had factor loadings ≤0.25 in the four dietary patters. Correlation values ≥0.3 or close to 0.3 (positive or negative) are highlighted in bold.
