# Supplementary material for: Emotional Eating and Dietary Patterns: Reflecting Food Choices in People with and without Abdominal Obesity
Source: Nutrients. 2022 Mar 25;14(7):1371. doi: 10.3390/nu14071371 (PMC9002960; doi:10.3390/nu14071371)
Supplement: Supplementary file 1 [file nutrients-14-01371-s001.zip › Supplementary Table S2.pdf]

Supplementary Table S2. Energy and nutrient intake according to non-adherence and adherence to each dietary patterns in participants with abdominal obesity

	Snacks and fast- food DP		Traditional Westernized DP		Healthy DP		Animal products and cereals DP	
	Non-adherence	Adherence	Non-adherence	Adherence	Non-adherence	Adherence	Non-adherence	Adherence
Energy (Kcal)	2058.4 ± 757.6	2616.4 ± 1097.4***	1920.4 ± 801.2	2754.4 ± 971.2***	2103.7 ± 770.4	2571.1 ± 1109.6***	2185.2 ± 953.6	2489.7 ± 989.1***
CH (g)	241.3 ± 95.3	311.1 ± 136.6***	229.7 ± 107.3	322.8 ± 119.7***	237.9 ± 89.7	314.5 ± 138.5***	270.8 ± 126.4	281.6 ± 119.0
Fiber (g)	20.3 ± 9.5	20.7 ± 9.9	19.3 ± 9.0	21.7 ± 10.2**	14.9 ± 5.6	26.1 ± 9.6***	19.7 ± 9.5	21.3 ± 9.8*
Proteins (g)	80.0 ± 31.9	92.7 ± 37.9***	74.2 ± 29.3	98.6 ± 37.1***	78.0 ± 30.1	94.7 ± 38.6***	74.2 ± 28.3	98.6 ± 37.9***
Lipids (g)	84.4 ± 33.4	110.2 ± 50.3***	80.1 ± 36.4	114.5 ± 45.5***	92.0 ± 37.8	102.6 ± 50.0**	88.2 ± 43.3	106.4 ± 44.1***
SFA (g)	22.1 ± 9.3	32.0 ± 15.2***	21.9 ± 10.5	32.2 ± 14.3***	27.6 ± 12.8	26.6 ± 14.3	23.5 ± 12.5	30.6 ± 13.7***
MFA (g)	29.6 ± 13.3	37.0 ± 17.1***	28.4 ± 13.8	38.1 ± 16.1***	30.4 ± 13.3	36.1 ± 17.4***	29.6 ± 14.2	36.9 ± 16.3***
PUFA (g)	18.4 ± 10.8	24.4 ± 14.5***	16.3 ± 9.7	26.6 ± 14.0***	20.4 ± 11.7	22.4 ± 14.4*	21.3 ± 13.8	21.5 ± 12.4
Cholesterol (mg)	337.4 ± 233.8	393.8 ± 267.6**	316.9 ± 255.9	414.3 ± 240.0***	351.5 ± 201.2	379.8 ± 294.9	284.0 ± 192.6)	447.3 ± 278.1***
Ethanol (g)	7.9 ± 18.0	8.7 ± 23.9	4.1 ± 7.6	12.5 ± 28.3***	6.7 ± 16.1	9.9 ± 25.1	7.3 ± 21.9	9.2 ± 20.4
Calcium (mg)	731.3 ± 279.2	927.0 ± 482.6***	717.3 ± 355.1	941.0 ± 422.9***	747.5 ± 380.9	910.8 ± 414.3***	762.4 ± 370.3	895.9 ± 429.0***
Phosphorus (mg)	1278.7 ± 485.9	1467.7 ± 593.1***	1192.6 ± 466.4	1553.8 ± 568.1***	1202.6 ± 442.3	1543.8 ± 593.0***	1224.9 ± 463.7	1521.5 ± 588.7***
Iron (mg)	19.0 ± 8.7	20.7 ± 8.9**	17.5 ± 8.3	22.2 ± 8.8***	17.0 ± 7.1	22.7 ± 9.5***	17.7 ± 7.8	21.9 ± 9.4***
Magnesium (mg)	413.3 ± 163.9	451.6 ± 179.0**	371.2 ± 145.5	493.7 ± 175.9***	352.5 ± 127.4	512.4 ± 174.9***	411.6 ± 158.0	453.2 ± 183.9**
Sodium (mg)	1522.8 ± 782.3	2255.7 ± 1194.9***	1598.4 ± 969.2	2180.1 ± 1095.5***	1851.6 ± 933.3	1926.9 ± 1198.1	1591.1 ± 1070.3	2187.4 ± 992.6***
Potassium (mg)	3625.2 ± 1452.7	3965.3 ± 1702.1**	3377.5 ± 1355.6	4213.1 ± 1696.3***	2999.6 ± 1001.0	4590.9 ± 1671.1***	3546.9 ± 1477.5	4043.6 ± 1660.9***
Zinc (mg)	9.4 ± 3.9	10.8 ± 4.8***	8.1 ± 2.9	12.2 ± 4.7***	9.2 ± 3.7	11.1 ± 4.8***	9.3 ± 3.8	11.0 ± 4.8***

Selenium (mcg)	37.5 ± 23.5	37.7 ± 20.7	32.9 ± 19.5	42.3 ± 23.6***	31.3 ± 14.3	43.9 ± 26.4***	32.8 ± 14.4	42.4 ± 27.0***
Vitamin A (mcg)	837.3 ± 484.7	948.4 ± 573.8**	831.7 ± 431.9	954.0 ± 613.5*	727.9 ± 461.9	1057.9 ± 550.0***	801.9 ± 428.5	983.8 ± 608.4***
Vitamin B1 (mg)	1.7 ± 0.7	2.0 ± 0.8***	1.5 ± 0.6	2.1 ± 0.8***	1.6 ± 0.6	2.1 ± 0.8***	1.7 ± 0.7	1.9 ± 0.8***
Vitamin B2 (mg)	2.6 ± 1.9	2.9 ± 1.5***	2.4 ± 1.3	3.2 ± 2.0***	2.5 ± 1.4	3.1 ± 2.0***	2.4 ± 1.3	3.2 ± 2.0***
Vitamin B3 (mg)	20.0 ± 8.7	22.9 ± 9.6***	18.7 ± 7.6	24.2 ± 9.9***	18.5 ± 7.1	24.4 ± 10.2***	19.0 ± 7.4	23.9 ± 10.3***
Vitamin B6 (mg)	1.9 ± 0.8	2.2 ± 1.0***	1.8 ± 0.8	2.3 ± 1.0***	1.7 ± 0.6	2.4 ± 1.0***	1.9 ± 0.9	2.2 ± 1.0***
Folate (mcg)	230.0 ± 110.9	259.6 ± 150.1**	234.6 ± 131.9	255.0 ± 132.9*	183.4 ± 74.0	306.3 ± 149.0***	222.0 ± 126.5	267.7 ± 135.0***
Vitamin B12 (mcg)	6.8 ± 4.2	7.4 ± 4.8	6.1 ± 3.5	8.1 ± 5.1***	6.3 ± 4.0	7.9 ± 4.9***	6.1 ± 3.6	8.1 ± 5.1***
Vitamin C (mg)	300.6 ± 205.9	303.0 ± 200.9	279.4 ± 179.2	324.2 ± 222.8*	204.0 ± 123.0	399.6 ± 220.0***	298.0 ± 205.1	305.6 ± 201.7
Vitamin E (mg)	0.8 ± 1.1	1.1 ± 1.3**	0.8 ± 1.0	1.1 ± 1.4**	0.8 ± 1.1	1.1 ± 1.3***	0.9 ± 1.3	1.0 ± 1.1*

DP: Dietary pattern; CH: Carbohydrates; MFA: Monounsaturated Fatty Acids; PUFA: Polyunsaturated Fatty Acids; SFA: Saturated Fatty Acids.
Data are presented as mean ± standard deviation. The log of all these variables was calculated for the statistical analysis; however, in this table we show the original mean values of the variables.
Differences between adherence and non-adherence to each dietary pattern were calculated by Student t-test with these transformed variables.
