# Supplementary material for: Emotional Eating and Dietary Patterns: Reflecting Food Choices in People with and without Abdominal Obesity
Source: Nutrients. 2022 Mar 25;14(7):1371. doi: 10.3390/nu14071371 (PMC9002960; doi:10.3390/nu14071371)
Supplement: Supplementary file 1 [file nutrients-14-01371-s001.zip › Supplementary Table S3.pdf]

Supplementary Table S3. Energy and nutrient intake according to non-adherence and adherence to each dietary patterns in participants without abdominal obesity

	**Traditional Westernized DP**		**Animal products, cereals and vegetables DP**		**Healthy DP**		**Snacks DP**	
	Non-adherence	Adherence	Non-adherence	Adherence	Non-adherence	Adherence	Non-adherence	Adherence
Energy (Kcal)	1950.9 ± 647.0	2744.1 ± 848.4***	2050.2 ± 785.7	2650.0 ± 811.2***	2191.9 ± 800.9	2507.3 ± 874.8***	2126.0 ± 819.2	2573.7 ± 827.3***
CH (g)	228.0 ± 86.3	316.8 ± 108.7***	239.9 ± 104.9	305.4 ± 100.4***	256.3 ± 106.8	288.9 ± 106.3**	233.2 ± 86.5	312.2 ± 112.4***
Fiber (g)	20.8 ± 9.4	21.1 ± 7.5	17.7 ± 7.3	24.2 ± 8.3***	17.2 ± 7.2	24.6 ± 8.1***	19.0 ± 7.7	22.9 ± 8.8***
Proteins (g)	79.8 ± 31.8	99.7 ± 37.1***	71.9 ± 19.9	107.9 ± 39.3***	84.4 ± 32.8	95.3 ± 38.2**	84.8 ± 35.1	94.9 ± 36.1**
Lipids (g)	81.4 ± 30.4	116.9 ± 40.9***	87.6 ± 38.5	110.9 ± 38.5***	92.3 ± 37.5	106.3 ± 41.6***	94.2 ± 43.4	104.3 ± 36.0**
SFA (g)	21.4 ± 8.3	34.9 ± 13.8***	24.5 ± 11.4	31.8 ± 13.9***	28.0 ± 13.9	28.3 ± 12.5	26.2 ± 14.2	30.2 ± 11.8***
MFA (g)	29.1 ± 13.8	40.9 ± 16.7***	31.3 ± 17.2	38.8 ± 14.7***	30.3 ± 12.5	39.8 ± 18.4***	34.1 ± 18.6	36.0 ± 13.8
PUFA (g)	17.4 ± 9.0	24.3 ± 12.2***	18.9 ± 11.5	22.9 ± 10.7***	19.5 ± 10.5	22.2 ± 11.9*	19.9 ± 11.1	21.9 ± 11.4
Cholesterol (mg)	357.7 ± 282.4	454.5 ± 265.8***	275.4 ± 118.6	538.1 ± 326.5***	418.1 ± 266.3	394.3 ± 289.7	438.2 ± 328.0	374.1 ± 212.6
Ethanol (g)	4.5 ± 7.7	10.4 ± 24.0***	7.2 ± 18.0	7.7 ± 18.2	5.8 ± 8.1	9.2 ± 24.2	6.8 ± 17.2	8.2 ± 18.9
Calcium (mg)	740.6 ± 302.9	967.3 ± 437.1***	711.2 ± 278.1	998.6 ± 436.5***	835.3 ± 447.2	873.6 ± 328.6	805.5 ± 430.9	903.6 ± 343.9***
Phosphorus (mg)	1310.4 ± 538.8	1568.3 ± 586.4***	1157.2 ± 344.1	1724.6 ± 623.1***	1336.0 ± 534.6	1544.5 ± 600.3***	1348.3 ± 571.2	1532.1 ± 569.7***
Iron (mg)	19.0 ± 8.5	22.8 ± 9.3***	16.4 ± 5.5	25.4 ± 9.8***	19.1 ± 9.3	22.6 ± 8.6***	19.8 ± 9.6	22.0 ± 8.5**
Magnesium (mg)	407.0 ± 154.9	470.9 ± 163.6***	373.6 ± 147.1	505.1 ± 150.0***	391.7 ± 143.4	486.8 ± 166.6***	413.4 ± 148.8	465.0 ± 171.4**
Sodium (mg)	1397.7 ± 617.5	2459.0 ± 1077.8***	1661.3 ± 929.7	2201.3 ± 1050.1***	1859.2 ± 1012.2	2001.9 ± 1038.5	1804.8 ± 1060.1	2056.8 ± 978.1**
Potassium (mg)	3671.2 ± 1390.1	4161.5 ± 1395.5***	3318.0 ± 1122.2	4521.0 ± 1419.7***	3406.3 ± 1221.5	4432.0 ± 1407.5***	3617.8 ± 1370.4	4218.9 ± 1393.4***
Zinc (mg)	8.7 ± 2.9	11.9 ± 4.5***	8.7 ± 3.3	11.9 ± 4.2***	9.8 ± 4.2	10.8 ± 3.9**	9.9 ± 4.1	10.7 ± 4.0*

Selenium (mcg)	36.6 ± 20.1	43.6 ± 22.0***	32.7 ± 14.2	47.6 ± 24.6***	34.8 ± 17.6	45.5 ± 23.4***	36.9 ± 20.2	43.3 ± 22.0**
Vitamin A (mcg)	881.7 ± 459.7)	1008.5 ± 516.7*	783.6 ± 395.2	1108.3 ± 527.1***	803.8 ± 396.7	1088.0 ± 537.6***	854.3 ± 428.5	1037.1 ± 535.3***
Vitamin B1 (mg)	1.6 ± 0.6	2.0 ± 0.7***	1.5 ± 0.5	2.1 ± 0.6***	1.7 ± 0.7	2.0 ± 0.6***	1.7 ± 0.6	2.0 ± 0.7***
Vitamin B2 (mg)	2.6 ± 1.8	3.4 ± 2.1***	2.3 ± 1.5	3.7 ± 2.2***	2.7 ± 1.4	3.3 ± 2.4**	2.8 ± 1.6	3.3 ± 2.3*
Vitamin B3 (mg)	20.4 ± 8.0	24.6 ± 9.2***	18.8 ± 6.2	26.3 ± 9.6***	19.7 ± 7.3	25.3 ± 9.5***	20.6 ± 7.7	24.5 ± 9.6***
Vitamin B6 (mg)	2.0 ± 0.8	2.3 ± 0.9***	1.8 ± 0.6	2.5 ± 0.9***	1.9 ± 0.7	2.4 ± 0.9***	1.9 ± 0.8	2.4 ± 0.9***
Folate (mcg)	253.6 ± 129.5	271.6 ± 112.4*	215.0 ± 91.8	310.5 ± 128.7***	208.8 ± 76.8	316.8 ± 133.5***	251.3 ± 120.9	274.0 ± 121.1*
Vitamin B12 (mcg)	7.4 ± 6.9	7.9 ± 5.0	5.6 ± 3.1	9.6 ± 7.4***	6.8 ± 4.1	8.4 ± 7.4	7.0 ± 6.4	8.3 ± 5.6*
Vitamin C (mg)	294.9 ± 159.7	298.0 ± 148.0	261.9 ± 150.3	331.2 ± 149.6***	238.0 ± 131.8	355.4 ± 151.9***	253.8 ± 128.9	339.4 ± 164.7***
Vitamin E (mg)	0.8 ± 0.9	0.9 ± 1.1	0.8 ± 0.9	0.9 ± 1.1	0.8 ± 0.9	1.0 ± 1.1	0.8 ± 0.9	0.9 ± 1.1

DP: Dietary pattern; CH: Carbohydrates; MFA: Monounsaturated Fatty Acids; PUFA: Polyunsaturated Fatty Acids; SFA: Saturated Fatty Acids.
Data are presented as mean ± standard deviation. The log of all these variables was calculated for the statistical analysis; however, in this table we show the original mean values of the variables.
Differences between adherence and non-adherence to each dietary pattern were calculated by Student t-test with these transformed variables.
*p<0.05, **p<0.01, ***p<0.001
