# Supplementary material for: Emotional Eating and Dietary Patterns: Reflecting Food Choices in People with and without Abdominal Obesity
Source: Nutrients. 2022 Mar 25;14(7):1371. doi: 10.3390/nu14071371 (PMC9002960; doi:10.3390/nu14071371)
Supplement: Supplementary file 1 [file nutrients-14-01371-s001.zip › Supplementary Table S4.pdf]

Supplementary Table S4. Energy and nutrient intake in participants with and without abdominal obesity

	**Total (n= 763)**	
	**Non-Abdominal Obesity**	**Abdominal Obesity**
Energy (Kcal)	2349.0 ± 851.8	2337.4 ± 982.5
CH (g)	272.6 ± 107.6	276.2 ± 122.7
Fiber (g)	20.9 ± 8.5	20.5 ± 9.7
Proteins (g)	89.8 ± 35.9	86.4 ± 35.6
Lipids (g)	99.2 ± 40.2	97.3 ± 44.6
SFA (g)	28.2 ± 13.2	27.1 ± 13.5
MFA (g)	35.0 ± 16.4	33.3 ± 15.7
PUFA (g)	20.9 ± 11.3	21.4 ± 13.1
Cholesterol (mg)	406.3 ± 277.9	365.6 ± 252.6
Ethanol (g)	7.5 ± 18.1	8.3 ± 21.2
Calcium (mg)	854.4 ± 392.4	829.2 ± 405.9
Phosphorus (mg)	1439.8 ± 576.8	1373.2 ± 549.8
Iron (mg)	20.9 ± 9.1	19.8 ± 8.9
Magnesium (mg)	439.1 ± 162.2	432.4 ± 172.5
Sodium (mg)	1930.3 ± 1026.0	1889.2 ± 1073.5
Potassium (mg)	3917.2 ± 1411.8	3795.3 ± 1589.9

Zinc (mg)	10.3 ± 4.1	10.1 ± 4.4
Selenium (mcg)	40.1 ± 21.3	37.6 ± 22.1
Vitamin A (mcg)	**945.4 ± 492.3**	**892.9 ± 533.5***
Vitamin B1 (mg)	1.8 ± 0.7	1.8 ± 0.8
Vitamin B2 (mg)	3.0 ± 2.0	2.8 ± 1.7
Vitamin B3 (mg)	**22.5 ± 8.9**	**21.4 ± 9.3***
Vitamin B6 (mg)	2.1 ± 0.9	2.0 ± 0.9
Folate (mcg)	**262.6 ± 121.3**	**244.8 ± 132.7****
Vitamin B12 (mcg)	7.6 ± 6.0	7.1 ± 4.5
Vitamin C (mg)	296.4 ± 153.6	301.8 ± 203.2
Vitamin E (mg)	0.9 ± 1.0	1.0 ± 1.2

CH: Carbohydrates; MFA: Monounsaturated Fatty Acids; PUFA: Polyunsaturated Fatty Acids; SFA: Saturated Fatty Acids.
Data are presented as mean ± standard deviation. The log of all these variables was calculated for the statistical analysis; however, in this table we show the original value of the variables.
Differences between those with non-abdominal obesity and abdominal obesity were calculated by Student t-test with these transformed variables ***p<0.05; **p<0.01**
