# Supplementary figures and images for: Emotional Eating and Dietary Patterns: Reflecting Food Choices in People with and without Abdominal Obesity
Source: Nutrients. 2022 Mar 25;14(7):1371. doi: 10.3390/nu14071371 (PMC9002960; doi:10.3390/nu14071371)

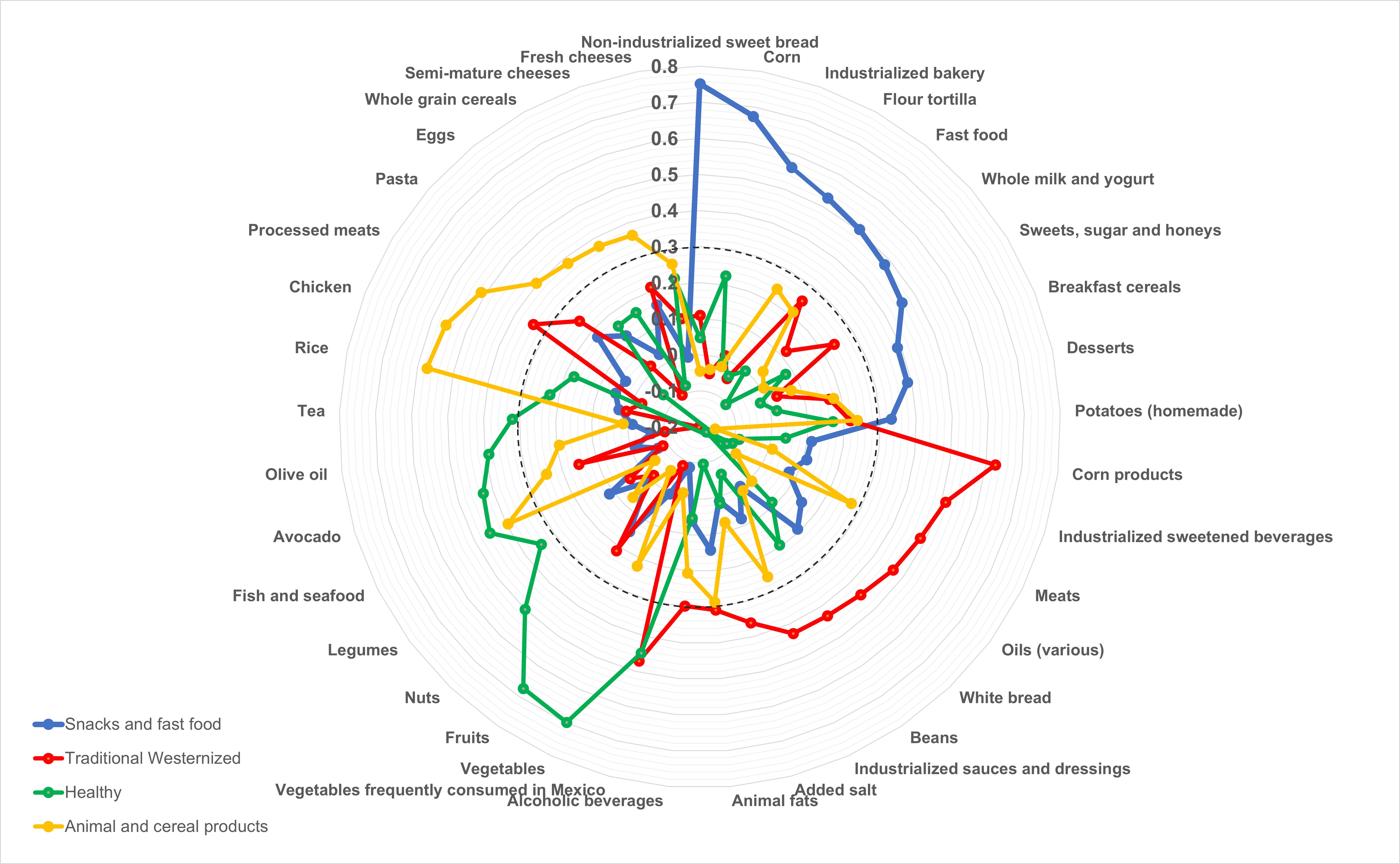

Supplement: Supplementary file 1 [file nutrients-14-01371-s001.zip › Supplementary Figure S1.jpg]
